# Binding of NF-κB to Nucleosomes: Effect of Translational Positioning, Nucleosome Remodeling and Linker Histone H1

**DOI:** 10.1371/journal.pgen.1003830

**Published:** 2013-09-26

**Authors:** Imtiaz Nisar Lone, Manu Shubhdarshan Shukla, John Lalith Charles Richard, Zahary Yordanov Peshev, Stefan Dimitrov, Dimitar Angelov

**Affiliations:** 1 Université de Lyon, Laboratoire de Biologie Moléculaire de la Cellule, CNRS-UMR 5239, Ecole Normale Supérieure de Lyon, Lyon, France; 2 Université Joseph Fourier - Grenoble 1, INSERM Institut Albert Bonniot, U823, Site Santé-BP 170, Grenoble, France; 3 Institute of Electronics, Bulgarian Academy of Sciences, Sofia, Bulgaria; Friedrich Miescher Institute for Biomedical Research, Switzerland

## Abstract

NF-κB is a key transcription factor regulating the expression of inflammatory responsive genes. How NF-κB binds to naked DNA templates is well documented, but how it interacts with chromatin is far from being clear. Here we used a combination of UV laser footprinting, hydroxyl footprinting and electrophoretic mobility shift assay to investigate the binding of NF-κB to nucleosomal templates. We show that NF-κB p50 homodimer is able to bind to its recognition sequence, when it is localized at the edge of the core particle, but not when the recognition sequence is at the interior of the nucleosome. Remodeling of the nucleosome by the chromatin remodeling machine RSC was not sufficient to allow binding of NF-κB to its recognition sequence located in the vicinity of the nucleosome dyad, but RSC-induced histone octamer sliding allowed clearly detectable binding of NF-κB with the slid particle. Importantly, nucleosome dilution-driven removal of H2A–H2B dimer led to complete accessibility of the site located close to the dyad to NF-κB. Finally, we found that NF-κB was able to displace histone H1 and prevent its binding to nucleosome. These data provide important insight on the role of chromatin structure in the regulation of transcription of NF-κB dependent genes.

## Author Summary

In eukaryotes DNA is hierarchically packaged into chromatin by histones. The fundamental subunit of chromatin is the nucleosome. The packaging of DNA into nucleosomes not only restricts DNA accessibility for regulatory proteins but also provides opportunities to regulate DNA based processes. Accessibility of transcription factor NF-κB to their recognition sequences embedded in nucleosomes is highly controversial. On one hand *in vivo* studies have suggested that packaging of DNA into chromatin plays an important role in regulating the expression of NF-κB dependent genes, and on the other hand some *in vitro* studies reported that NF-κB can bind by itself to its recognition sequences embedded in the nucleosome. In this study, we show that NF-κB can specifically bind to its recognition sequences placed at the end of the nucleosome but not when placed inside the nucleosome core. We then demonstrate that disruption of nucleosome is necessary for the productive binding of NF-κB. Finally, we show that the presence of histone H1 does not affect the specific binding of NF-κB to its cognate sequence, when its binding region overlaps with the binding site of NF-κB. We propose that histone eviction is needed for NF-κB to bind specifically to its recognition sequence embedded in the nucleosome.

## Introduction

In eukaryotes, all DNA-templated reactions occur in the context of chromatin. The repeating structure of chromatin, the nucleosome, consists of a nucleosome core (made up of two copies of each core histone H2A, H2B, H3 and H4) around which 147 bp of DNA is wrapped [1], a linker histone and a linker DNA [2]. The packaging of promoter DNA in nucleosomes inhibits transcription *in vitro* [3] and *in vivo* [4]. Sequence-specific binding of transcription factors is the key event for gene activation. Promoters of repressed genes, however, are usually embedded in nucleosomes. To activate gene expression, transcription factors must get access to their regulatory sites. In general the nucleosomes represent a barrier for the access of transcription factors to their cognate sequence [5]. Some transcription factors such as human glucocorticoid receptor [6], [7], [8], yeast PHO2/PHO4 proteins [9], and GAL4 [10], [11] have been shown to bind to their recognition sequences embedded in the nucleosomes. The binding of some of these transcription factors was found to be dependent on the length of the recognition sequence and on both the recognition sequence distance from the nucleosomal ends and its rotational orientation [12]. Binding of disparate transcription activators is inherently cooperative, which highlights the fact that disruption of nucleosomes by one factor (Gal4) is sufficient to allow the binding of another factor (NF-κB) at the interior of the nucleosome [12]. Other distinct transcription factors, such as Sp1, Lef-1, ETS-1 and USF have also been shown to be able to invade the nucleosome and to interact with their cognate sequences [13], [14].

The key regulator of gene expression in inflammation is the family of transcription factors NF-κB/Rel [15]. NF-κB/Rel are sequence-specific DNA-binding proteins [16] that initiate transcription [17] from a variety of genes that are involved in immune response and inflammatory processes [18], [19], [20]. Ways to modulate the levels of these transcription factors in inflammation and cancer are considered to be of potential therapeutic importance [21], [22]. In mammalian cells, the NF-κB/Rel family contains five members: RelA (p65), c-Rel, RelB, NF-κB1 (p50; p105) and NF-κB2 (p52; p100) [23]. p50 and p52 usually form homodimers or heterodimers with one of the other three proteins. Each type of NF-κB homo- and hetero-dimer has both slightly different DNA-binding affinity and specificity for κB sites bearing different variations of the consensus sequence GGGRNNYYCC (R is purine, Y is pyrimidine and N is any base), but nonetheless their functions often overlap [24], [25], [26], [27]. These dimers are kept in the cytoplasm in an inactive form by interaction with a class of inhibitory proteins called as inhibitory kappa B [23]. This cytoplasmic inhibition provides a way to regulate the activation of the NF-κB dimers. Once activated, NF-κB dimers translocate to nucleus and bind to their cognate sites in target genes and initiate their transcription. Apart from the cytoplasmic regulation, NF-κB dependent genes are also regulated inside the nucleus by the local chromatin structure [28]. Depending on the chromatin organization, some NF-κB binding sites are found in a constitutively accessible state; many others are occluded by nucleosomes and hence, depend on additional regulatory mechanisms at the level of chromatin. Although, several studies have made it clear that proper expression of NF-κB-dependent genes requires dynamic cross-talk of promoters and enhancers with the local chromatin environment [29], [30], [31], [32], [33], [34] it still remains elusive whether NF-κB binding is a prerequisite for chromatin remodeling or whether local chromatin settings dictate NF-κB binding activities to trigger selective expression of a specific gene.

There is extensive evidence that nucleosomes interfere with NF-κB recruitment *in vivo* [28], [34], 35. It has been suggested that positioned nucleosomes could be selectively remodeled upon the stimulation of the cells by LPS treatment to make the nucleosomes accessible to restriction enzymes [36]. Especially, secondary response genes as well as the primary response genes induced with delayed kinetics were shown to be dependent on SWI/SNF mediated remodeling of the nucleosmes [34].

Binding of NF-κB to its recognition sequence placed inside the nucleosome is dependent on nucleosome remodeling was also shown in an *in vitro* study [37]. It was reported that NF-κB does not bind to its recognition sequence placed 30 bp away from the end. However, remodeling of these nucleosomes by SWI/SNF allowed NF-κB binding [37]. Although these studies highlighted the role of chromatin in regulating the accessibility of κB sites, some *in vitro* studies provided contradictory evidence. For example, it was reported that the recognition site located 39 bp from the nucleosomal end was fully accessible to NF-κB (p50 homodimer) and only partially accessible when located 52 bp from the end of the nucleosomal DNA [13]. Another study claimed, however, that NF-κB p50 homodimer was able to invade the nucleosome and to bind to its recognition sequence independent of its localization relative to the end of the nucleosome core particle [38]. Both these studies lead to a controversy as they rule out the role of epigenetic regulators such as chromatin remodelers in regulating the initial binding of NF-κB to NF-κB-dependent genes. Besides, the x-ray crystal structure of the distinct NF-κB-DNA complexes show that the κB sites are almost encircled by NF-κB dimers [39], [40], [41], [42]. Such a specific mode of binding would not be achieved if the κB sites are wrapped in nucleosomes owing to steric hindrances. Moreover, if NF-κB is able to bind its recognition sequence wrapped in nucleosomes, it would be incompatible with the demonstrated role of remodelers in activation of subset of NF-κB dependent genes [33].

The reported *in vitro* studies on the binding of transcription factors in general and of NF-κB in particular have some serious limitations. First, the sequences that were used for nucleosome reconstitution do not allow the generation of a homogenous population of positioned nucleosomes as these sequences have relatively weak nucleosome positioning potential [43]. Second, the studies were carried out generally at low nucleosome concentration where the nucleosomes are unstable and subnucleosomal particles might be formed through H2A–H2B removal [13], [38]. Third, in several cases the techniques used to probe the binding of transcription factors did not have sufficient resolution in order to make a firm conclusion about the specificity of the binding. In addition, how does the presence of histone H1 and ATP-dependent nucleosome remodelers affect the transcription factor binding has not been studied in details.

In this work we have overcome these limitations by using both strongly centrally positioned nucleosomes together with a combination of EMSA, hydroxyl radical (•OH) footprinting and UV laser footprinting to analyze how the histone octamer, histone H1 and remodeling and mobilization of the nucleosome impact the binding of NF-κB to its recognition sequence. Our data suggests a possible *in vivo* mechanism of NF-κB binding and transcriptional regulation of inflammatory NF-κB responsive genes.

## Results

### Characterization of the nucleosomal templates used to study NF-κB binding

**Figure 1:**
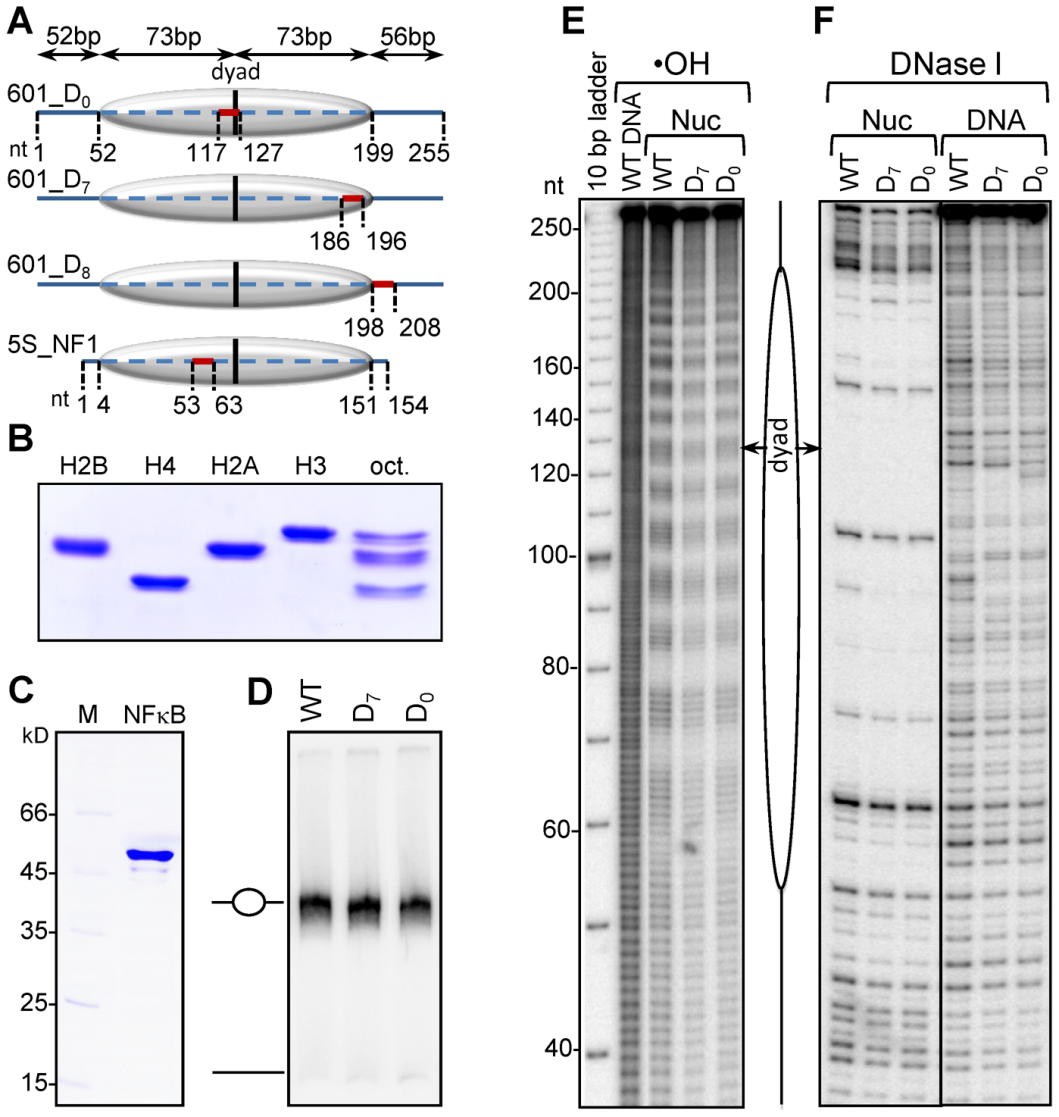
Characterization of the reconstituted nucleosomes. (**A**) Schematics of the reconstituted nucleosomes. The canonical NF-κB site was inserted in the 255 bp 601 DNA fragment either at the dyad of the nucleosome (601_D_0_ DNA) or at the nucleosomal end (601_D_7_ DNA) or in the linker DNA (601_D_8_ DNA); bold lines, free DNA arms; dashed line, core particle region. The vertical black line represents the dyad. The NF-κB binding sites (BS) are depicted by the red line. The length of each region is shown on top of the constructs. The very bottom schematics shows the location of the NF-κB binding site inserted in the 5S rDNA fragment of *Xenopus borealis* used for nucleosome reconstitution. (**B**) Electrophoretic analysis of the indicated purified recombinant histones and histone octamer. (**C**) Electrophoretic analysis of purified recombinant NF-κB (p50); M, molecular marker; p50, the p50 subunit of NF-κB. (**D**) Nucleosome reconstitution check by 5% native PAGE. (**E**) •OH radical and (**F**) DNase I footprinting of free 601 DNA and the indicated reconstituted nucleosomes.

To study the interaction NF-κB (p50 homodimer) with the nucleosome we have used recombinant proteins purified to homogeneity (Figure 1 B and C). In order to have a homogenous population of nucleosomes, we used a highly reliable and standard synthetic nucleosome positioning sequence 601 derived by Lowary and Widom solely based on its highest affinity and ability to position nucleosomes [44]. We also used a naturally occurring and relatively lower affinity nucleosome positioning sequence derived from somatic 5S RNA gene of *Xenopus Borealis* [43]. Centrally positioned nucleosomes were reconstituted on either 255 bp 601 DNA fragment or on 152 bp 5S rDNA (Supplementary figure S1). Since NF-κB p50 homodimer exhibits clear affinity for continuous G stretches [26] ( see supplementary Figure S2), some of the G's within G-rich regions of the 601 DNA were substituted with either T or A (See Supplementary Figure S1), which decreased the less-specific associations of NF-κB. To study the effect of translational positioning of the binding site on the NF-κB binding, MHC-H2 κB site was inserted either in the vicinity of nucleosome dyad (601_D_0_) or at the edge of the nucleosome (601_D_7_). Furthermore, to study the effect of linker histone H1 on the binding of NF-κB, MHC-H2 κB site was inserted at the binding region of H1 (601_D_8_). In the case of 5S rDNA the MHC-H2 NF-κB site was inserted close to the dyad (Figure 1A and supplementary Figure S1).

EMSA shows that under the experimental conditions, all the DNA was reconstituted into nucleosomes (Figure 1D). The reconstituted 601 particles exhibit clear 10 bp repeat upon cleavage with either •OH radicals (Figure 1E) or with DNase I (Figure 1F). This was evidence for both proper wrapping of DNA around the histone octamer and strong octamer positioning relative to the DNA ends in the reconstituted particles. We conclude that the reconstituted nucleosomes represent a very homogenous population of particles suitable for further NF-κB-nucleosome binding studies.

### Terminal segments of the nucleosomal DNA, but not sequences located at vicinity to the nucleosome dyad, are accessible to NF-κB

**Figure 2:**
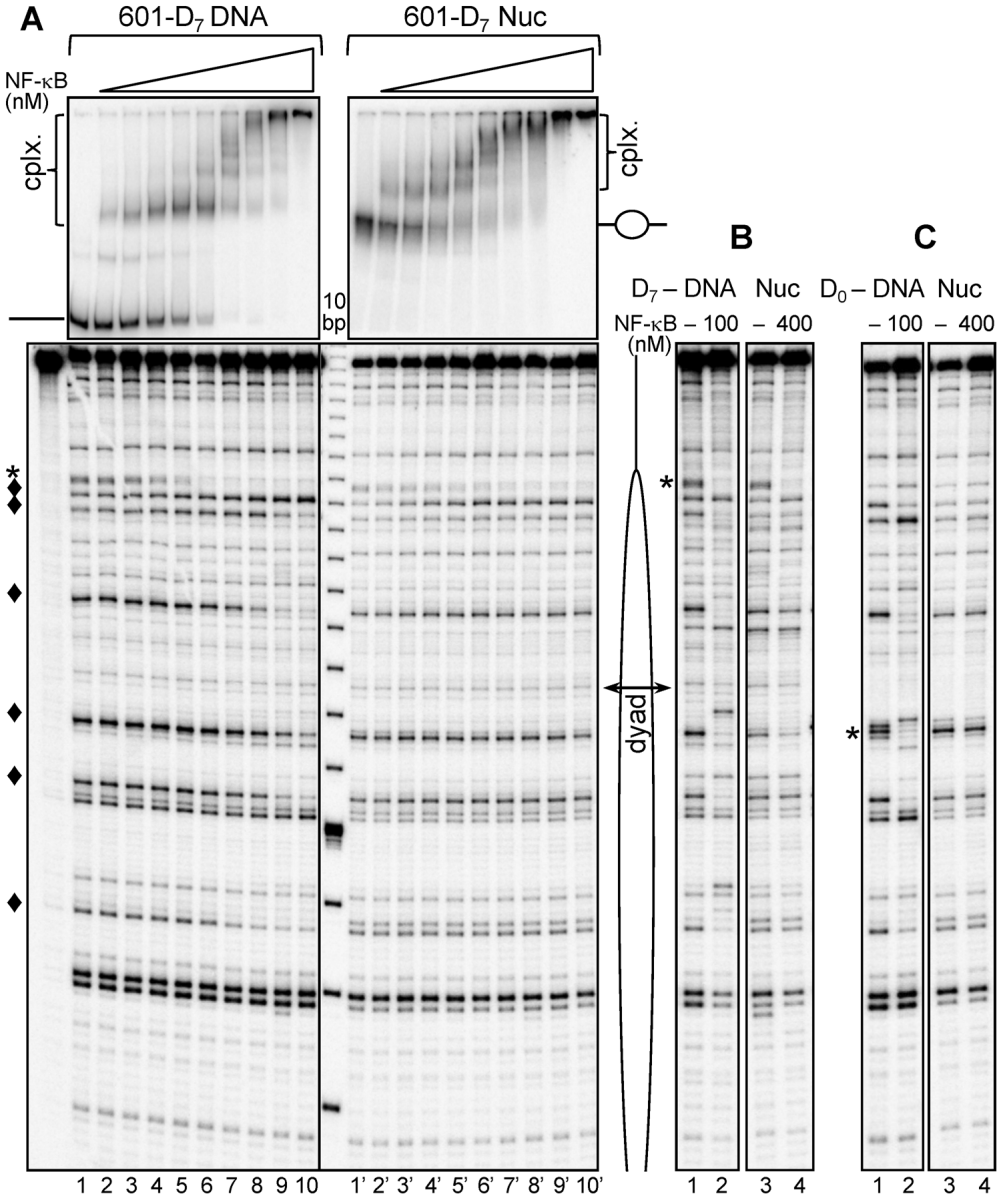
NF-κB binds specifically at the nucleosomal ends, but not at the nucleosomal dyad. (**A**) **Upper panel**: EMSA of NF-κB binding to naked 601_D_7_ DNA (left) or to 601_D_7_ nucleosome (right). Naked ^32^P-end labeled 601_D_7_ DNA or nucleosomes were incubated with increasing amount of NF-κB (1.5 fold serial dilution of NF-κB was performed starting with saturating amounts of 100 nM for naked DNA and 400 nM for nucleosome). The aliquots of the reaction mixtures were run on a 5% native PAGE. The positions of free DNA and nucleosomes are indicated, “cplx.” represents NF-κB – DNA/nuc complexes; **lower panel:** UV laser footprinting patterns of the NF-κB-DNA and NF-κB-nucleosomes complexes. The respective remaining mixtures were irradiated with a single 5 nanoseconds UV laser 266 nm pulse (*E*_pulse_∼0.1 J/cm^2^), DNA was purified from the samples and then treated with Fpg glycosylase. The cleaved DNA fragments were separated on 8% sequencing gel and visualized by autoradiography; (*), NF-κB footprint at the high-affinity NF-κB binding site; (♦), NF-κB footprints at low-affinity sites. A schematic presentation of the nucleosomes is shown on the right side; the double headed arrow indicates the nucleosomal dyad. (**B**) UV laser footprinting of NF-κB bound to either naked 601_ D_7_-DNA (lanes 1 and 2) or to 601_D_7_-nucleosome (lanes 3 and 4) repeated with saturated amounts of NF-κB as indicated. (**C**) Same as (B), but for naked 601_D_0_ DNA and 601_D_0_-nucleosomes. Note the absence of specific NF-κB footprint at the dyad in the case of the nucleosome.

To study the effect of translational positioning of NF-κB recognition sequence within the nucleosomes, we analyzed the binding of NF-κB by using both EMSA and UV laser footprinting (Figure 2). EMSA shows that incubation of either free 601_D_7_ DNA or 601_D_7_ nucleosomes with increasing amount of NF-κB results in the generation of several bands with lower electrophoretic mobility (Figure 2A). These bands are due to the binding of either one or several NF-κB molecules. However, since only one high affinity NF-κB binding sequence is present within the templates, only one band should reflect the specific NF-κB binding to this site, while the others would reflect either the NF-κB binding to lesser affinity sites (G-rich regions) or non-specific interaction of NF-κB with the templates.

The analysis of the UV laser footprinting pattern and EMSA data shows that these three types of NF-κB binding are present in the NF-κB/DNA complexes. Indeed, the decrease and disappearance of the G-specific bands corresponding to high affinity MHC-H2 κB site (designated by *) parallels the appearance and the rise of the first shift in EMSA (Figure 2A, upper left panel). This indicates that NF-κB is mostly bound specifically to this site. Moreover, the appearance of additional bands in EMSA correlates with the change in the intensity of the other G-specific bands (designated by ♦) in the UV laser footprints, reflecting that additional molecules of NF-κB are binding to low affinity GC-rich sites. Besides, the incubation of DNA with NF-κB at high concentrations results in the generation of additional super-shifts in EMSA without alterations in the UV laser footprinting pattern (Figure 2A), which is obviously determined by the completely non-specific association of additional NF-κB molecules to DNA. Therefore, the combination of the UV laser footprinting with EMSA allows the visualization of both the binding of NF-κB to high and low affinity sites (see also the quantification presented in supplementary figure S2) and a clear detection of the non-specific binding.

In the case of nucleosomes, the behavior of NF-κB binding is, however, different (Figure 2). Indeed, the presence of histone octamer interferes with the NF-κB binding efficiency since 4 times more NF-κB has to be present in the reaction mixture in order to observe comparable band shifts (Figure 2A, upper panel). To check whether NF-κB binds specifically to its cognate sequence located at the edge of the nucleosome, we performed the UV laser footprinting. The results show that NF-κB binds specifically to its cognate sequence located at the edge of the nucleosome as evidenced by the decreasing intensity of the band originating from the MHC-H2 NF-κB sequence in the footprinting pattern (Figure 2A, lower panel) which parallels the appearance of the first shift in EMSA (Figure 2A, upper panel). No footprinting of the low affinity sites (designated by ♦) located inside the nucleosome core particle is observed and thus, the histone octamer “shields” these low affinity sites and prevents NF-κB from binding to them. All these results suggest that nucleosome core DNA is inaccessible to NF-κB. To further analyze this we inserted a high affinity MHC-H2 κB site at the center of the nucleosome core (Figure 1A) and asked if NF-κB binds to this high affinity site or not. As expected, binding of NF-κB to a high affinity sequence inserted close to the dyad in the 601_D_0_ nucleosome, was not achieved even at 400 nM concentration of NF-κB (Figure 2C). These results clearly demonstrate that nucleosomes act as a barrier to NF-κB binding irrespective of the affinity of the binding site.

### Binding of NF-κB to remodeled and to slid 601_D_0_ nucleosomes

Nucleosome remodeling is an essential principle to assure that the compact eukaryotic genomes in chromatin remains flexible and adaptable to regulatory needs [45]. The RSC chromatin remodeler is able to both remodel and slide centrally positioned nucleosomes [46]. RSC uses a stepwise mechanism for nucleosome remodeling. During the first step a stable non-mobilized particle is generated that contains ∼180 bp DNA loosely associated with the histone octamer [46]. This particle, termed “remosome,” can be further mobilized by RSC. The histone-DNA interactions within the remosome are perturbed and these perturbations allow accessibility of restriction enzymes all along the remosomal DNA [46]. The RSC-remodeled products, namely remosomes and slid nucleosomes, can be fractioned by native PAGE (see [46] and schematics in Supplementary Figure S3).

**Figure 3:**
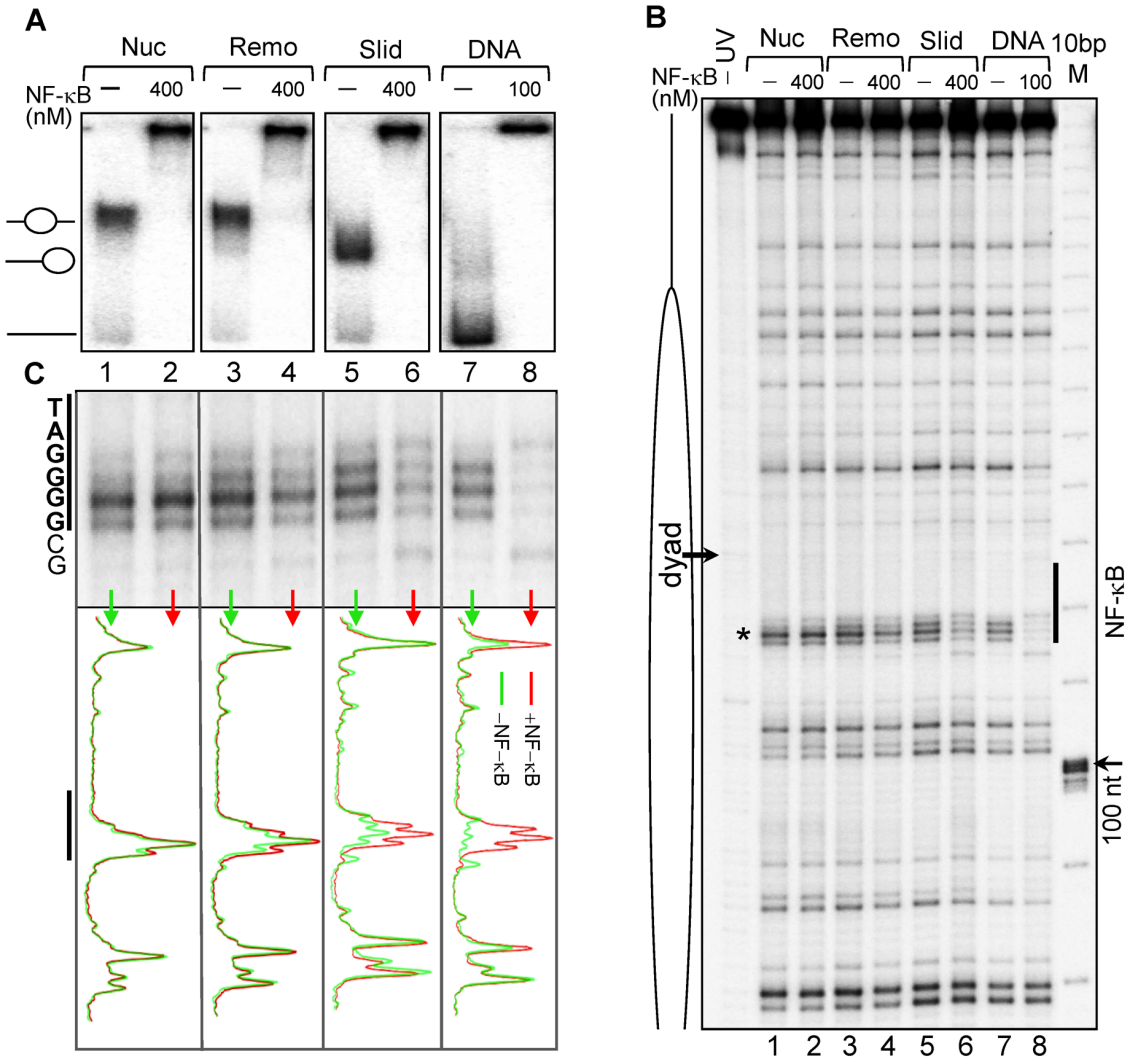
Binding of NF-κB to remodeled and slid nucleosomes. (**A**) Nucleosomes, RSC-remodeled nucleosomes (remosomes), slid nucleosomes and naked 601_D_0_ DNA were incubated with the indicated amount of NF-κB and separated on a 5% native PAGE. The positions of the different particles are shown on the left part of the gel. (**B**) UV laser footprinting of the indicated distinct NF-κB bound particles. The experiment was carried out as described in Figure 2. The NF-κB binding site is shown as vertical black line and the black arrow indicates the nucleosomal dyad; M, 10 bp DNA molecular marker (**C**) top, “zoom” of the NF-κB binding region from the footprints shown in (B); bottom, scan of the footprints; red, in presence NF-κB; green in absence of NF-κB.

Does the generation of remosomes or nucleosome mobilization permit binding of NF-κB to the 601_D_0_ nucleosome? To test this we have prepared control centrally positioned 601_D_0_ nucleosomes and both non-mobilized remodeled nucleosome (remosomes) and slid (end-positioned) nucleosomes. Then we have studied the binding of NF-κB to these templates by using UV laser footprinting. Control EMSA shows that NF-κB is able to associate with all templates at the NF-κB concentrations used (Figure 3A). However, the RSC-induced perturbations in the histone-DNA interactions were not sufficient to allow specific binding of NF-κB to the 601_D_0_ remosomes, since the observed changes in the photoreactivity (≤10%) were very low and unspecific (Figure 3B and 3C, lanes 3 and 4). However, the NF-κB-slid nucleosome complex exhibits detectable alterations (∼40–50% fraction) in the footprinting pattern characteristic of specific binding (Figure 3B and 3C, compare lanes 5 and 6). Note that these alterations are not as strong as those observed in the footprinting pattern of naked DNA (Figure 3B, lanes 7 and 8). We attributed this to the fact that upon nucleosome sliding no more than 50% of the potentially available NF-κB binding sites will be localized sufficiently close to the nucleosomal ends (see Figure 1). Only these repositioned nucleosomes will be accessible to NF-κB.

### Removal of H2A–H2B dimer from the nucleosome allows specific NF-κB binding to the nucleosomal dyad

**Figure 4:**
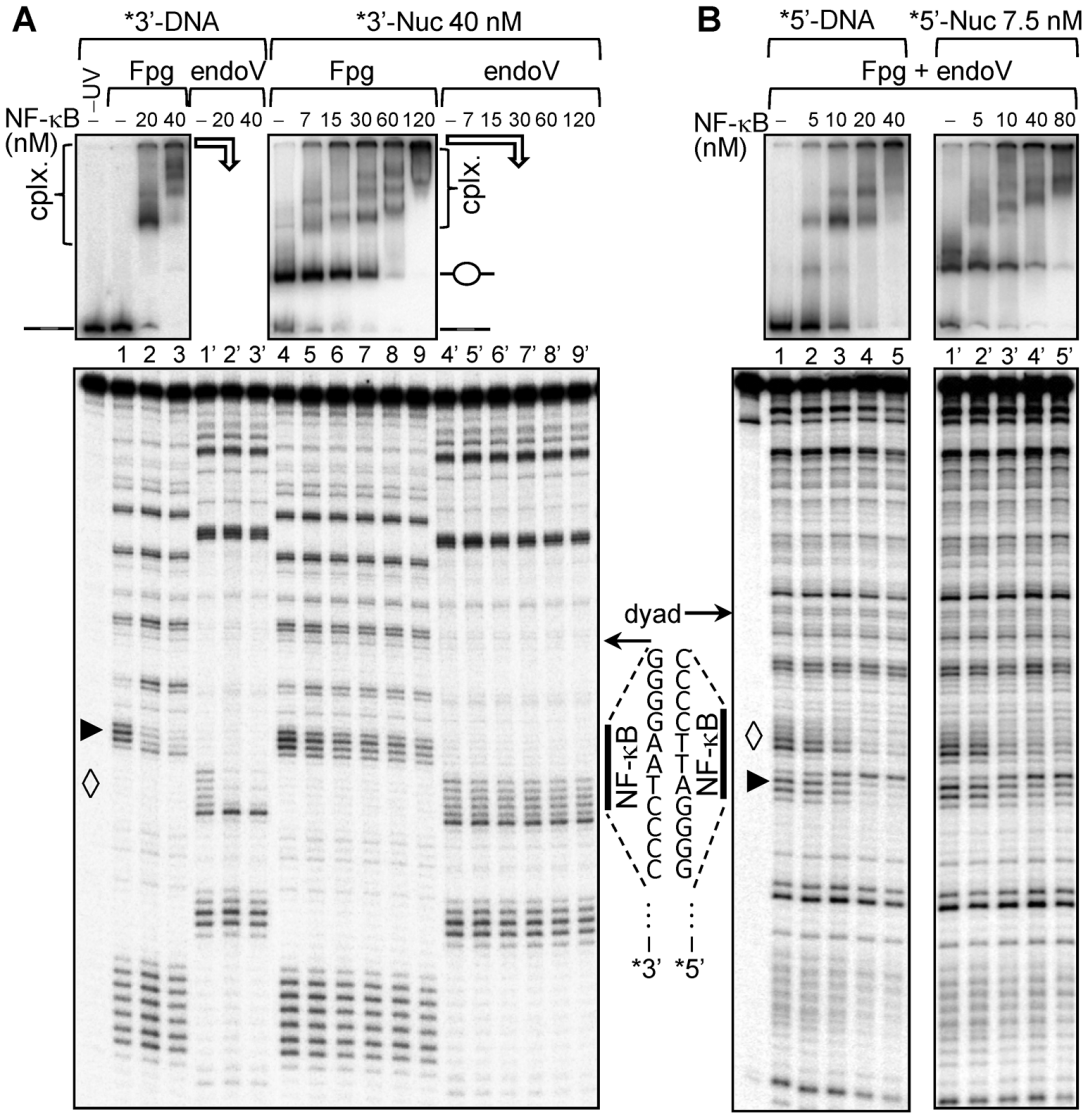
Dilution driven H2A–H2B dimer eviction allows binding of NF-κB to Nucleosome Core Particle. (**A**) 152 bp DNA fragment derived from *X. borealis* somatic 5 S RNA gene containing single NF-κB site near the dyad NF1 (53–56) was amplified by PCR and uniquely 3′ end labeled with α-^32^P by Klenow. Nucleosomes were reconstituted on this labeled fragment as described previously. The DNA and nucleosomes at a concentration 40 nM were incubated with increasing amounts of NF-κB as indicated to allow the formation of stable complexes which were subsequently irradiated by a single high intensity UV laser pulse (*E*_pulse_∼0.1 J/cm^2^). The formation of complexes was checked by EMSA (upper panel, DNA lane 1–3, nucleosomes lane 4–9), the positions of free DNA and nucleosomes are indicated, “cplx.” represents NF-κB – DNA/nuc complexes. The samples were split into two parts, DNA was purified and treated with Fpg glycosylase to cleave 8-oxoG (represented by ▸,lower panel, DNA lane 1–3 and nucleosome lane 4–9) and with T4 endonuclease V to cleave CPDs (represented by ◊, DNA lane 1′–3′ and nucleosome lane 4′–9′). The cleaved DNA fragments were visualized by 8% sequencing gel. (**B**) The same 152 bp 5S fragment was 5′ end labeled with γ-^32^P by T4 polynucleotide kinase and used for nucleosome reconstitution. DNA and nucleosomes, at 10 nM final concentration, were incubated with increasing amounts of NF-κB as indicated to allow the formation of complexes. The assembly of the complexes was checked by EMSA (upper panel, DNA lane 1–5, nucleosomes lane 1′–5′). The samples were irradiated with a single high intensity UV laser pulse (*E*_pulse_∼0.1 J/cm^2^), treated with a mix of Fpg glycosylase and T4 endonuclease V to cleave both the 8-oxoG (▸) and CPDs (◊). Finally, the cleaved products were visualized by 8% sequencing gel (DNA, lane 1–5; nucleosomes lane 1′–5′). The NF-κB binding sites (vertical bold lines) and the NF-κB recognition sequences are shown. The arrows designated the nucleosomal dyad.

As shown above, the histone octamer in both the nucleosomes and the remosomes impeded NF-κB binding to its cognate sequence inserted in the vicinity of the dyad of the 601 nucleosome core particles. This could reflect at least in part the higher stability of the particles reconstituted with the 601 sequence. To test this possibility, we reconstituted nucleosome core particles using the lower affinity 5S rDNA containing the NF-κB cognate sequence close to the dyad (5S_NF1 nucleosome, see Figure 1A) and next asked if NF-κB could bind to it. The experiments were carried out similarly to those performed with the 601_D_7_ nucleosome (Figure 4). The binding reactions were carried out at 40 nM nucleosome concentration (at this concentration the whole histone octamer is associated with DNA) and aliquots of the reaction mixtures were analyzed by EMSA. The formation of complexes were clearly seen (Figure 4 A, upper panels). The remaining samples were submitted to UV laser footprinting and treated with either Fpg glycosylase or with T4 Endonuclease V. In contrast to the naked DNA-NF-κB complexes, no specific change in the footprinting pattern of the NF-κB binding site within the NF-κB-nucleosome complexes was detected (Figure 4A, lower panel). The slight proportional change in the band intensity observed within the G-repeat (lanes 4–9) did not resemble the respective specific changes in naked DNA (lanes 1–3) and hence did not qualify as specific binding. Moreover, no detectable change was observed in the pyrimidine tract of the binding site (compare lanes 1′–3′ with lanes 4′–9′). These results, combined with the EMSA data, demonstrate that NF-κB associates in a non-specific manner with the nucleosome core particle although some preference for the purine half-binding site might exist. Therefore, the lower stability of the 5S rDNA nucleosome was not sufficient to allow specific NF-κB binding suggesting that more drastic structural perturbations of the nucleosomes might be required.

To test this possibility, we prepared nucleosomes lacking one or two H2A–H2B dimers by simple dilution of 5S_NF1 nucleosomes. Indeed, at about 10 nM nucleosome concentration, H2A–H2B dimers partially dissociate from the nucleosome (Supplementary figure S4) without affecting the positioning of the remaining (H3-H4)_2_ histone tetramer relative to the ends of nucleosomal DNA [47], [48]. Bearing this in mind, we diluted 5S nucleosome core particles to ∼7.5 nM concentration and then incubated them with increasing amount of NF-κB (Figure 4B). At this nucleosomal concentration two populations were observed in the migration of control nucleosomes (Figure 4 upper panels, compare lane 4 in panel A with lane 1′ in panel B) suggesting that nucleosomes were perturbed. EMSA shows that NF-κB formed complexes with all the studied templates (Figure 4B, upper panels). However, in contrast to the 5S_NF1 nucleosome-NF-κB complexes formed at 40 nM concentration (where the H2A–H2B dimers are bound to nucleosomal DNA), a very well pronounced and specific footprinting pattern in both the purine and the pyrimidine runs (compare lanes 1–5 with lanes 1′–5′ in Figure 4B lower panel) was observed in 5S_NF1 nucleosomes-NF-κB complexes at 7.5 nM concentration (H2A–H2B dimers partially removed). We conclude that eviction of one or two H2A–H2B dimers is essential for the specific binding of NF-κB to its cognate site located in nucleosome core.

### Effect of histone H1 on the NF-κB specific interaction with nucleosomal DNA

Histone H1 is an essential player in modulating and maintaining chromatin architecture [49], [50]. In contrast to core histones, it consists of three domains, a structured (“globular”) domain and unstructured and lysine rich N- and C-termini. The globular domain of histone H1 interacts with both the nucleosome dyad and two short (10 bp) sequences at the very beginning of each of the two linker DNAs. The C-terminus of H1 binds to the remaining linker DNA, brings together the two linkers and forms the “stem” like structure and thus leads to chromatin compaction [51], [52].

**Figure 5:**
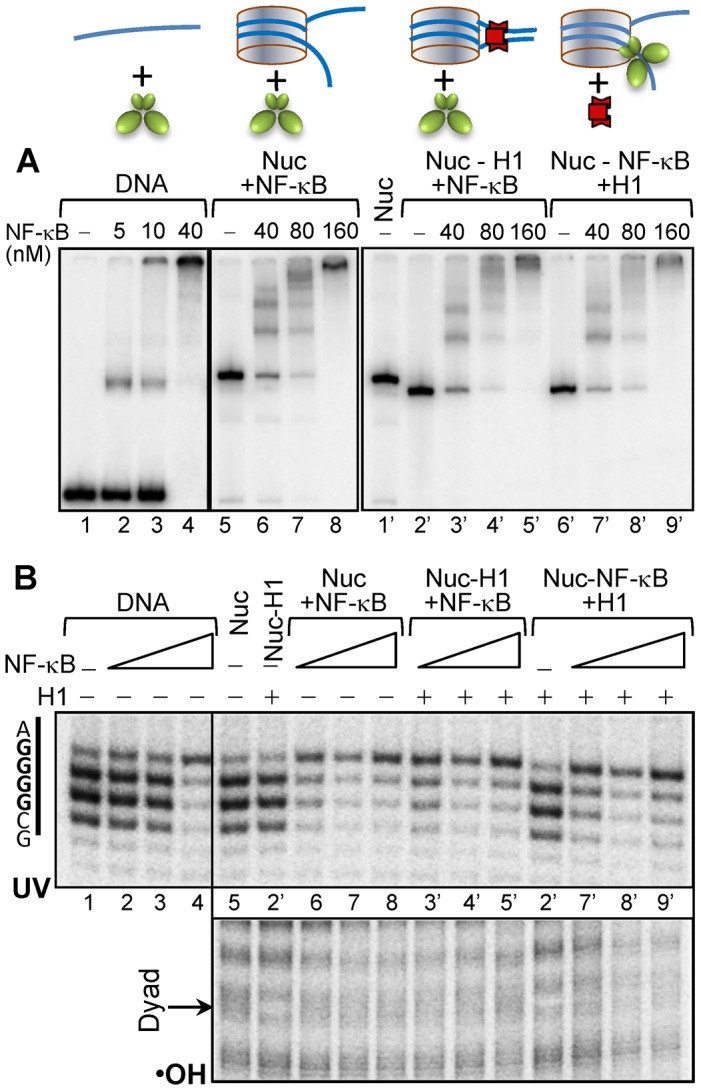
NF-κB displaces H1 from the chromatosome and binds to its recognition sequence. (**A, upper panel**), schematics of the substrates used in each experiment. (**A, lower panel**), EMSA of the binding of NF-κB to depicted substrates. The bottom strand of the 255 bp 601_D_8_ DNA (Supplementary figure S1) was uniquely 5′-end labeled by ^32-^P and used to reconstitute centrally positioned nucleosomes. Chromatosomes were assembled by using the NAP-1/H1 complex to properly deposit H1 on the nucleosome in H1/nuc ratio of ∼1.5. The first two panels show the NF-κB-DNA (lanes 1–4) and NF-κB-nucleosome (lanes 5–8) complexes formed upon incubation with increasing amount of NF-κB. The last panel illustrates both the interaction of chromatosomes with the indicated increasing amount of NF-κB (lanes 1′–5′) and the deposition of H1 on the already assembled (at increasing NF-κB concentration) NF-κB nucleosome complexes (lanes 6′–9′). (**B**) UV laser (upper panel) and •OH (lower panel) footprinting of the NF-κB binding region of NF-κB-DNA and distinct NF-κB-nucleosome complexes.

To analyze how H1 affects the interaction of NF-κB with the nucleosomes, we constructed 601_D_8_ nucleosomes in which the recognition sequence of NF-κB completely overlapped with the binding region of the globular domain of histone H1 on one of the linker DNA (see Figure 5A and Figure 1A). We then used NAP-1 to deposit H1 properly on the nucleosomes [52]. Next, we asked if NF-κB has access to its binding site by using EMSA, •OH and UV-laser footprinting (Figure 5 and supplementary Figure S5). The combination of these three approaches allows to judge for the overall association of NF-κB with the nucleosome (EMSA), the presence of H1 on the nucleosome (•OH footprinting) and the specific binding of NF-κB to its cognate sequence (UV laser footprinting). EMSA shows that NF-κB associates with all used naked DNA and nucleosomal templates and that increase of the concentration of NF-κB leads to the formation of particles containing more than one NF-κB molecules (Figure 5A). Interestingly, binding of NF-κB to naked DNA and nucleosome both with and without H1 gave rise to a very clear UV laser footprinting, thus demonstrating that NF-κB is able to invade the H1 containing nucleosome (Figure 5B, upper panel). The presence of H1, in agreement with the reported data [52], resulted in a very clear •OH footprint at the nucleosomal dyad (Figure 5B, lower panel). This footprint disappeared upon binding of NF-κB to the nucleosome, thus suggesting an NF-κB induced removal of H1 (Figure 5B, lower panel). Notably, when NAP-1-H1 was added to the 601_D_8_ nucleosome-NF-κB complex, no removal of NF-κB by NAP-1-H1 was observed (Figure 5B, lower panel). Therefore, in contrast to the core histones, H1 can be displaced by the binding of NF-κB and once NF-κB is bound, adding of H1 does not affect the stability of the 601_D_8_ nucleosome complexes.

## Discussion

Most studies of gene induction by inflammatory stimuli have focused on transcription factors that recognize specific DNA sequences and the cytoplasmic events that regulate the activation of these transcription factors. However, transcriptional activation of eukaryotic genes is also influenced by chromatin structure. Studies on the alterations in the chromatin structure required for productive NF-κB binding are essential for understanding the control of expression of inflammatory genes. However, the available data on this important topic are scarce and contradictory [15]. Here we used a combination of EMSA, ^•^OH and UV laser footprinting to analyze how NF-κB binds to nucleosomes and the effect of histone H1 on the binding. Our data provide definitive evidence that NF-κB is able to bind specifically to its cognate sequence when inserted at the end of the nucleosome, but not when it was inserted in vicinity to the nucleosome dyad. The accessibility to the ends of the nucleosome could be explained by the weaker histone-DNA interactions at these sites and their spontaneous unwrapping [53], [54]. At the center (the dyad) of the nucleosome, where the histone-DNA interactions are very strong, NF-κB is unable to specifically bind its cognate site. By contrast, several studies in the past have reported that some transcription factors, including NF-κB, were able to invade the nucleosome and to bind to nucleosome-embedded recognition sequences even when located in the center of nucleosomal DNA [13], [38]. However, these studies were carried out at low nucleosome concentrations at which sub-nucleosomal (hexameric & tetrameric) particles tend to appear [47], [48]. In order to understand whether the nucleosomes per se act as barriers for transcription factor binding, it is imperative to have homogenous population of nucleosomes devoid of any sub-nucleosomal entities. These sub-nucleosomal entities are formed by the loss of one or both H2A–H2B dimers and hence contain disorganized nucleosomal DNA which most likely would permit the specific binding of transcription factors. Our results demonstrate that eviction of H2A/2B dimers is required for the binding of NF-κB. This can be achieved by the binding of factors that can disrupt the nucleosomes either directly by themselves as shown by Adams and Workman for Gal4 [12] or indirectly through the recruitments of other nucleosome disrupting activity.

It has also been reported that the remodeling of 156 bp nucleosome core particles by SWI-SNF leads to complete and specific binding of NF-κB to its binding sites buried inside the nucleosome [37]. However, we observed only partial binding of NF-κB at the nucleosomal dyad when the nucleosomes are repositioned by the ATP dependent remodeler RSC. This discrepancy could be attributed to partial histone eviction [55] under the very high concentration of the SWI-SNF that was used to remodel the nucleosomes and/or a presumable instability of the non-canonical (loss of the dyad axis) slid core particles as reported by Bartholomew group [56], [57].

Unexpectedly, in contrast to the partial accessibility to dimeric restriction enzymes at the dyad and efficient base excision repair (BER) initiation [58], [59] remosomes did not show specific binding of NF-κB. Thus, in line with the available structural information [39], [41], [42], the specific binding of NF-κB requires much higher perturbations in histone-DNA interactions and unpeeling of its cognate sequence from the histone surface allowing it to “embrace” DNA and to productively bind to it. Our experimental results further demonstrate that such specific and productive binding could be efficiently achieved when the H2A–H2B dimer is removed from the nucleosome or when the histone octamer is repositioned in a way that the binding site nears the edge.

The compaction of chromatin by the linker histone in general has a global and repressive impact on transcription. Linker histone H1 brings the two helices close to each other and leads to the formation of a so-called ‘stem’ structure [52]. Binding of H1 to DNA at the termini of nucleosomes inhibit spontaneous wrapping and unwrapping of DNA and hence would prevent the binding of transcription factors. Another possibility in which H1 could affect transcription is by occupying the binding sequences of those transcription factors whose binding sites are located in the linker region. This suggests that TF will have to compete with H1 to bind to their cognate sites. Several studies have provided the evidence that in certain cases linker histone can be directly displaced by transcription factor [49], [60], [61]. In agreement with these studies, we found that the presence of histone H1 does not prevent the specific binding of NF-κB when their binding regions overlap. In fact, NF-κB binding completely displaces histone H1 from the nucleosomes. We also observed that H1 cannot displace the specifically bound NF-κB. *In vivo*, this competition might be even more in favor of NF-κB as H1 is quite mobile and dynamic [62].

Our *in vitro* data sheds light on the *in vivo* requirements for the alterations in chromatin structure necessary for the productive binding of NF-κB. These include either a removal of H2A–H2B dimers from the nucleosome and/or chromatin remodeler induced mobilization of the histone octamer. The H2A–H2B dimers are more easily displaced from the histone core than H3 and H4 [63], [64] and they are extensively exchanged *in vivo* [65]. Moreover, in mammalian cells the nucleosomes in the vicinity of the TSS contain the histone variant H2A.Z [66], [67], [68]. A tentative hypothesis is that specific chaperones, recognizing variant H2A.Z nucleosomes, could be involved in the removal of H2A.Z–H2B variant dimer, thus allowing binding of the NF-κB transcription factors to any site of the nucleosomal DNA.

## Materials and Methods

### Preparation of DNA fragments

The 255 bp 601 DNA from pGEM-3Z-601.1 (kindly provided by J. Widom) was modified in order to insert the MHC-H2 NF-κB binding sites at various positions and to remove the potential nonspecific κB sites. The DNA fragments were cloned into PCR2.1-TOPO and amplified by PCR. End labeling of the fragments was done by using ^32^P labeled primers. To generate the 5S 152 bp fragments carrying the NF-κB binding site, 213 bp *Xenopus Borealis* 5S gene fragment was amplified by PCR from the modified pXP10 vector. This fragment was digested with EcoR1; end labeled using either polynucleotide kinase [γ-^32^P] ATP or klenow fragment [α-^32^P] ATP and finally digested with Rsa1 to generate 152 bp fragments. To label the bottom strand, the 213 bp fragment was first digested with Rsa1 and end labeled by polynucleotide kinase [γ-^32^P] followed by digestion with EcoR1. The MHC-H2 κB site (ggggattcccc) is introduced in the pXP10 vector between −16 to −26, 5S_NF1 as described in [38]. All labeled DNA substrates were purified on 5% native acryl amide gel prior to use for nucleosome reconstitutions. Details of the sequences are shown in the supplementary figure S1.

### Proteins expression and purification

Recombinant *Xenopus laevis* full-length core histones (H2A, H2B, H3, and H4) were expressed in the form of inclusion bodies in E. coli strain BL21 (DE3) and purified as described in [69]. Remodels Structure of Chromatin (RSC) complex was purified essentially as described previously [70]. A clone (PET3-H1.5) encoding full-length 227 amino acid residue human H1.5 was expressed by standard IPTG induction in transformed BL21- RIL bacterial cells. The soluble proteins were purified first by SP sepharose and then by fractionation over a 1-mL Resource S cation exchange column (Biorad) using FPLC [52]. Mouse NAP-1 (mNAP-1) was also bacterially expressed and purified by Resource Q anion exchange column. Purified proteins were analyzed by 15% SDS-PAGE and stained with Coomassie blue. NF-κB p50 homodimer was prepared as mentioned in [71].

### Nucleosome reconstitution

Nucleosome reconstitution was performed by the salt dialysis procedure [72]. Approximately, 200 ng of ^32^P-labeled DNA probe containing the NF-κB binding site and 2.3 µg of chicken erythrocyte DNA (150–200 bp) as carrier were mixed with histone octamer, approximately in 1∶1 ratio in nucleosome reconstitution buffer (2 M NaCl, 10 mM Tris, pH 7.4, 1 mM EDTA, 5 mM 2-Mercaptoethanol) and the mixture was dialyzed from 2 M NaCl to 10 mM NaCl overnight at 4°C.

### NF-κB binding reaction

The binding reaction of NF-κB and DNA or nucleosomes was carried out at 37°C. Typically, NF-κB was mixed with DNA or nucleosome in a 20 µl reaction containing 1× binding buffer (10 mM Tris, pH 7.4, 75 mM NaCl, 1 mM EDTA, 1 mM DTT, 100 µg/ml BSA, 0.01% NP40 and 5% glycerol). In nucleosome dilution experiments NP40 was omitted from the buffer. The naked DNA was supplemented with carrier nucleosomes to a final concentration equal to those of labeled nucleosomes (≈40 nM). An aliquot of this reaction mix was used to check the formation of the NF-κB/DNA or NF-κB/nucleosome complex by 5% native PAGE at room temperature in 0.3× Tris-borate-EDTA (TBE) buffer. The remaining aliquots were probed by UV laser footprinting. Essentially the electrophoretic mobility shift assay and the UV laser footprinting were done on the same reaction.

### Remodeling of nucleosomes by RSC and remosome purification

Fractionation of remodeling nucleosomal species was performed as described in [46]. Typical remodeling reactions were performed with 1 pmol of nucleosomes and ≈40 fmol of RSC in remodeling buffer (10 mM Tris pH 7.4, 5% glycerol, 1 mM rATP, 2.5 mM MgCl_2_, 1 mM DTT, 100 µg/mL BSA, 50 mM NaCl, 0.01% NP40) in a volume of 10 µL at 29°C for 45 minutes. Under these conditions ∼50% of nucleosomes are translocated to the DNA extremities (see supplementary Figure S3) and the slow-migrating band contains essentially remosomes and a very low amount of intact nucleosomes (see Figure 5C in [46]). The reaction was stopped by addition of 0.02 units of apyrase and 1 µg of plasmid DNA. The products of remodeling and the control nucleosomes were run on 5% native gel. The bands corresponding to control nucleosome, remosome and slid nucleosome were excised and eluted in 80 µl elution buffer EB, containing 10 mM Tris pH 7.4, 0.25 mM EDTA, 10 mM NaCl, 0.01% NP40 and 30 nM chicken erythrocytes nucleosomes (to avoid dilution of nucleosomes) at 4°C for 3 hours (Supplementary Figure S3). Eluted nucleosomes were filtered through glass fiber filter under low speed centrifugation (200×g) to remove acryl amide particles and slightly concentrated to 50 µl volume using 100 KDa cutoff centricons. The gel eluted DNA, nucleosomes, remosomes and slid nucleosomes were used for NF-κB binding study. Typically saturating amounts of NF-κB (DNA: 100 nM, Nucleosome, remosome and slid nucleosome: 400 nM) were added to each excised species in 1× binding buffer. An aliquot of this reaction was used to check the formation of complex by EMSA and the remaining aliquot was used for UV laser footprinting.

### UV laser footprinting

The UV laser footprinting is based on change in the UV laser induced nucleotide photoreactivity upon protein binding [73], [74]. Irradiation of protein-DNA complexes by a single UV laser pulse results in different nucleotide lesions, whose spatial distribution depends on the type of proteins specifically bound to the DNA [74]. Quantitative measurements of the lesions and comparison with those of free DNA allows the analysis (at single-base resolution) of the changes in the structure of DNA upon protein binding. The use of UV lasers has many advantages compared to conventional light sources. With a single UV laser pulse a footprint of the protein is achieved. Additionally, in contrast to conventional light sources, high intensity laser irradiation induces specific biphotonic oxidative lesions in DNA (in addition to monophotonic pyrimidine dimers) [74]. These lesions are extremely sensitive to local DNA structure and can be easily mapped by enzymatic DNA strand cleavage followed by electrophoresis under denaturing conditions [75], [76]. In our study we have mapped the UV laser specific biphotonic lesions 8-oxoG by Fpg glycosylase and the monophotonic cyclobutane pyrimidine dimers (CPDs) by T4 Endonuclease V cleavage, both generated in the NF-κB cognate sequence upon UV laser irradiation.

The samples were exposed to a single high intensity UV laser pulse (E_pulse_∼0.1 J/cm^2^) as described in [74], [75]. The DNA was then purified by phenol-chloroform and ethanol/glycogen precipitated. The purified DNA was resuspended in resuspension buffer (10 mM Tris, pH 7.4, 30 mM NaCl, 1 mM EDTA, 1 mM DTT, 100 µg/ml BSA, 0.01% NP40) and cleaved with 0.1 units of either Fpg glycosylase or T4 endonuclease V (Trevigen) or both. The DNA was lyophilized and resuspended in formamide loading buffer, heated for 3 minutes at 80°C and loaded on 8% sequencing gel in 1× TBE buffer. The gels were dried and exposed overnight on a phosphor imager screen. The screens were scanned on phosphor imager and analyzed by Multi-Gauge (Fuji) software.

### NAP-1 mediated deposition of H1

Full-length human H1 was mixed with mNAP-1 in a 1∶2 molar ratio (buffer 20 mM Tris-HCl, pH 7.5, 0.5 mM EDTA, 100 mM NaCl, 1 mM DTT) and incubated at 30°C for 15 minutes. This mix of Nap-1/H1 was added to the NF-κB/nucleosome binding reaction either before or after addition of NF-κB in experimentally determined ratio in order to achieve 100% incorporation (see Supplementary Figure S6). Each reaction was subjected to buffer exchange by means of 30 KDa cutoff centricon concentrators so that to have H1/nucleosomes/NF-κB complexes in a ^•^OH quencher free buffer (5 mM Tris, 5 mM NaCl, and 0.25 mM EDTA) for hydroxyl radical footprinting. After the buffer exchange, each reaction was split into three parts; one part was analyzed by electrophoretic mobility shift assay (EMSA) in 5% native PAGE to check the formation of the complexes. The second part was analyzed by hydroxyl radical footprinting to visualizing the H1 binding and third part was analyzed by UV laser footprinting to probe the NF-κB binding.

### Hydroxyl radical footprinting

Hydroxyl footprinting was carried out in 15 µl final reaction mixture in quencher free buffer placed at the bottom of an eppendorf tube. The hydroxyl radicals were generated by mixing 2.5 µl each of 2 mM FeAmSO_4_/4 mM EDTA, 0.1 M ascorbate, and 0.12% H_2_O_2_ together in a drop on the side of the reaction tube before mixing rapidly with the reaction solution. The reaction was terminated after 2 minutes by addition of 100 µL stop solution (0.1% SDS, 25 mM EDTA, 1% glycerol, and 100 mM Tris, pH 7.4), and the DNA was purified by phenol/chloroform extraction and ethanol/glycogen precipitation. The DNA was resuspended in formamide loading buffer, heated for 3 minutes at 80°C and run on 8% denaturing gel in 1× TBE buffer. The gels were dried and exposed overnight on a phosphor imager screen. The gels were scanned on phosphor imager and analyzed by Multi-Gauge Fuji.

## Supporting Information

Figure S1 Sequences of the different 255 bp 601 DNA fragments and 152 bp 5S rDNA used for nucleosome reconstitution. (A) The sequence of the 255 bp 601 nucleosomal DNA. The bold region represents the 147 bp nucleosome core, nucleosome dyad is in red. (B) The sequence of the modified 255 bp 601 nucleosomal DNA with the NF-κB binding site inserted at the dyad of the nucleosome. The MHC-H2 NF-κB binding site is in uppercase and highlighted in grey. The substitution of Gs by either T or A are in blue and underlined. (C) The sequence of the modified 255 bp 601 nucleosomal DNA with the NF-κB binding site inserted at the edge of the nucleosome. Green highlighted g's represent the low affinity NF-κB binding sites marked as diamonds in Figure 2A. (D) The sequence of the 255 bp modified 601 nucleosomal DNA with the NF-κB binding site inserted in the linker DNA starting from the nucleosome edge. (E) 154 bp 5S core particle DNA sequence.
(TIF)

Figure S2 601_D7 DNA containing the MHC-H2 binding site was analyzed for NF-κB binding. This experiment was done essentially in the same way as in Figure 2. Naked ^32^P-end labeled 601_D_7_ DNA was incubated with increasing amount of NF-κB as indicated. (A, upper panel) The aliquots of the reaction mixtures were analyzed by 5% native PAGE (EMSA). The positions of free DNA and Its complexes with NF-κB are indicated. (A, lower panel) UV laser footprinting of the NF-κB-DNA complexes. The UV laser irradiated and Fpg glycosylase cleaved DNA fragments were separated on 8% sequencing gel and visualized by autoradiography. Apparent binding constants for MHC-H2 site (K_d_ = 14 nM) and other region displaying less affinity but still specific binding of NF-κB are displayed in the right side. (B) Quantified sequencing gel data plots used for determining of apparent binding constants.
(TIF)

Figure S3 Remosome purification assay. (A) Schematic: Naked DNA, nucleosomes and the remodeling reaction products (remosomes and slid nucleosomes) were run on 5% polyacrylamide gel. The corresponding bands (1, 2, 3, and 4) were cut and the substrates were eluted from the gel as described in material and methods. The purified substrates were then allowed to bind saturating amount of NF-κB (100 nM for DNA and 400 nM for other substrates) and submitted to UV laser footprinting. The formation of complexes with NF-κB is analyzed by 5% polyacrylamide gel. (B) The original preparative 5% PAGE.
(TIF)

Figure S4 H2A/H2B dimer loss upon nucleosome dilution. Nucleosome dilution experiment was performed by using radioactive labeled histone H3* and H2B* by Aurora kinase B and [γ-^32^P]ATP as described in [47]. These labeled histones together with other recombinant histones were used to reconstitute H2B* labeled or H3* labeled nucleosomes. Aliquots of nucleosomes were diluted with the appropriate buffer (75 mM NaCl) in a 20 µl final volume to the concentrations indicated (from 50 to 2.5 nM) and left for 45 min at room temperature. Then the samples were analyzed by electrophoretic mobility shift assay carried out in 5% polyacrylamide gel in 0.3× TBE at 4°C.
(TIF)

Figure S5 Hydroxyl radical and UV laser footprinting of NF-κB–DNA/nucleosome/chromatosome complexes. 255 bp 601_D_8_ DNA was ^32-^P end labeled and used to reconstitute centrally positioned nucleosomes. Chromatosomes were assembled by using the NAP-1/H1 complex to deposit H1 on the nucleosome under “physiological” conditions. Complete gel of the experiment shown in figure 5 for the overall comparison and analysis of UV laser footprinting (lanes 1–16) and •OH footprinting (lanes 5′–16′) (for details see figure 5).
(TIF)

Figure S6 SDS PAGE of nucleosomes and chromatosomes. Reconstituted nucleosomes and chromatosomes were analyzed on 18% SDS gel for verifying the histone composition after the buffer exchange for hydroxyl radical footprinting.
(TIF)
