# Supplementary figures and images for: Binding of NF-κB to Nucleosomes: Effect of Translational Positioning, Nucleosome Remodeling and Linker Histone H1
Source: PLoS Genet. 2013 Sep 26;9(9):e1003830. doi: 10.1371/journal.pgen.1003830 (PMC3784511; doi:10.1371/journal.pgen.1003830)

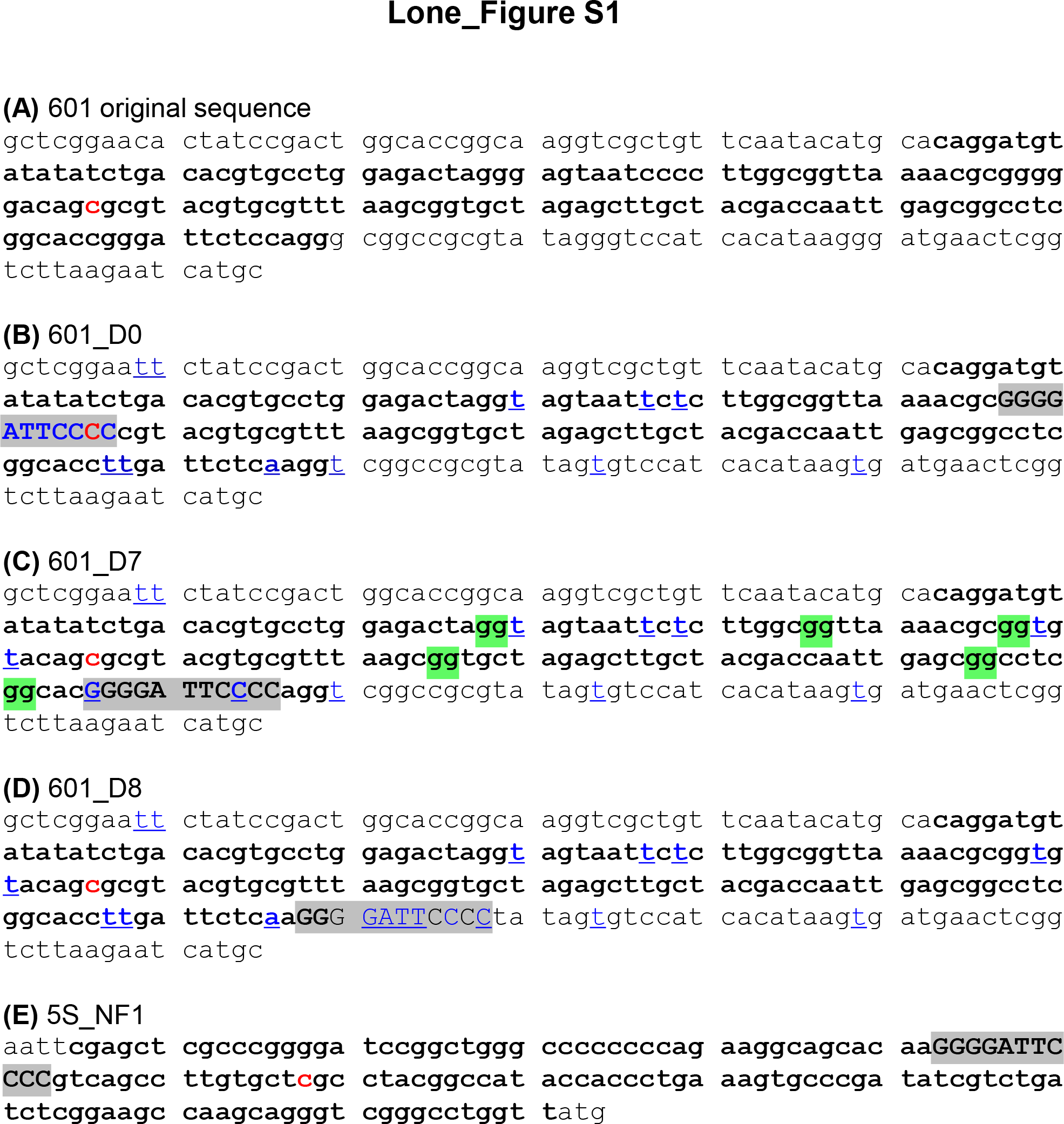

Supplement: Figure S1 — Sequences of the different 255 bp 601 DNA fragments and 152 bp 5S rDNA used for nucleosome reconstitution. (A) The sequence of the 255 bp 601 nucleosomal DNA. The bold region represents the 147 bp nucleosome core, nucleosome dyad is in red. (B) The sequence of the modified 255 bp 601 nucleosomal DNA with the NF-κB binding site inserted at the dyad of the nucleosome. The MHC-H2 NF-κB binding site is in uppercase and highlighted in grey. The substitution of Gs by either T or A are in blue and underlined. (C) The sequence of the modified 255 bp 601 nucleosomal DNA with the NF-κB binding site inserted at the edge of the nucleosome. Green highlighted g's represent the low affinity NF-κB binding sites marked as diamonds in Figure 2A. (D) The sequence of the 255 bp modified 601 nucleosomal DNA with the NF-κB binding site inserted in the linker DNA starting from the nucleosome edge. (E) 154 bp 5S core particle DNA sequence. (TIF) [file pgen.1003830.s001.tif]

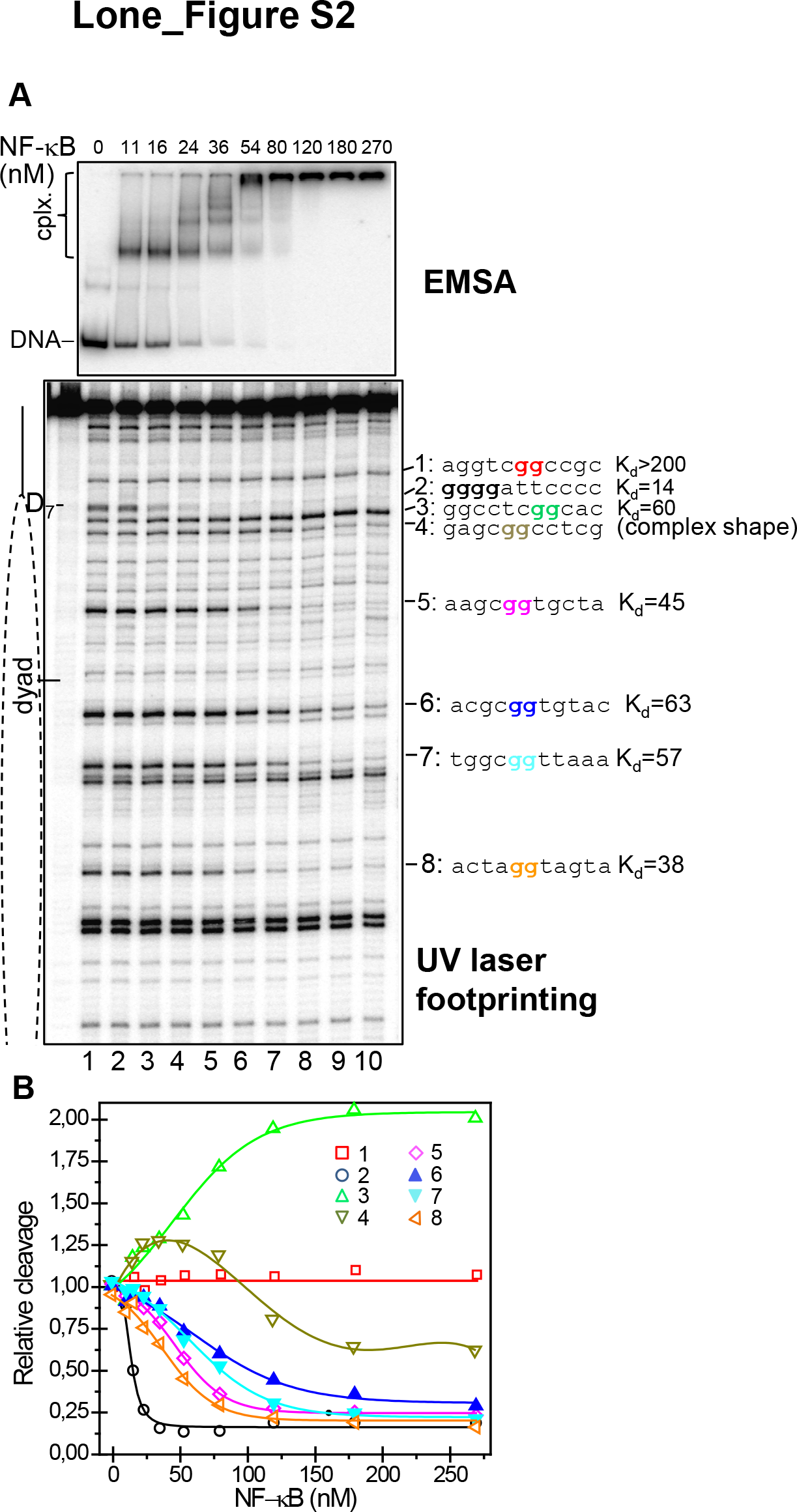

Supplement: Figure S2 — 601_D7 DNA containing the MHC-H2 binding site was analyzed for NF-κB binding. This experiment was done essentially in the same way as in Figure 2. Naked 32P-end labeled 601_D7 DNA was incubated with increasing amount of NF-κB as indicated. (A, upper panel) The aliquots of the reaction mixtures were analyzed by 5% native PAGE (EMSA). The positions of free DNA and Its complexes with NF-κB are indicated. (A, lower panel) UV laser footprinting of the NF-κB-DNA complexes. The UV laser irradiated and Fpg glycosylase cleaved DNA fragments were separated on 8% sequencing gel and visualized by autoradiography. Apparent binding constants for MHC-H2 site (Kd = 14 nM) and other region displaying less affinity but still specific binding of NF-κB are displayed in the right side. (B) Quantified sequencing gel data plots used for determining of apparent binding constants. (TIF) [file pgen.1003830.s002.tif]

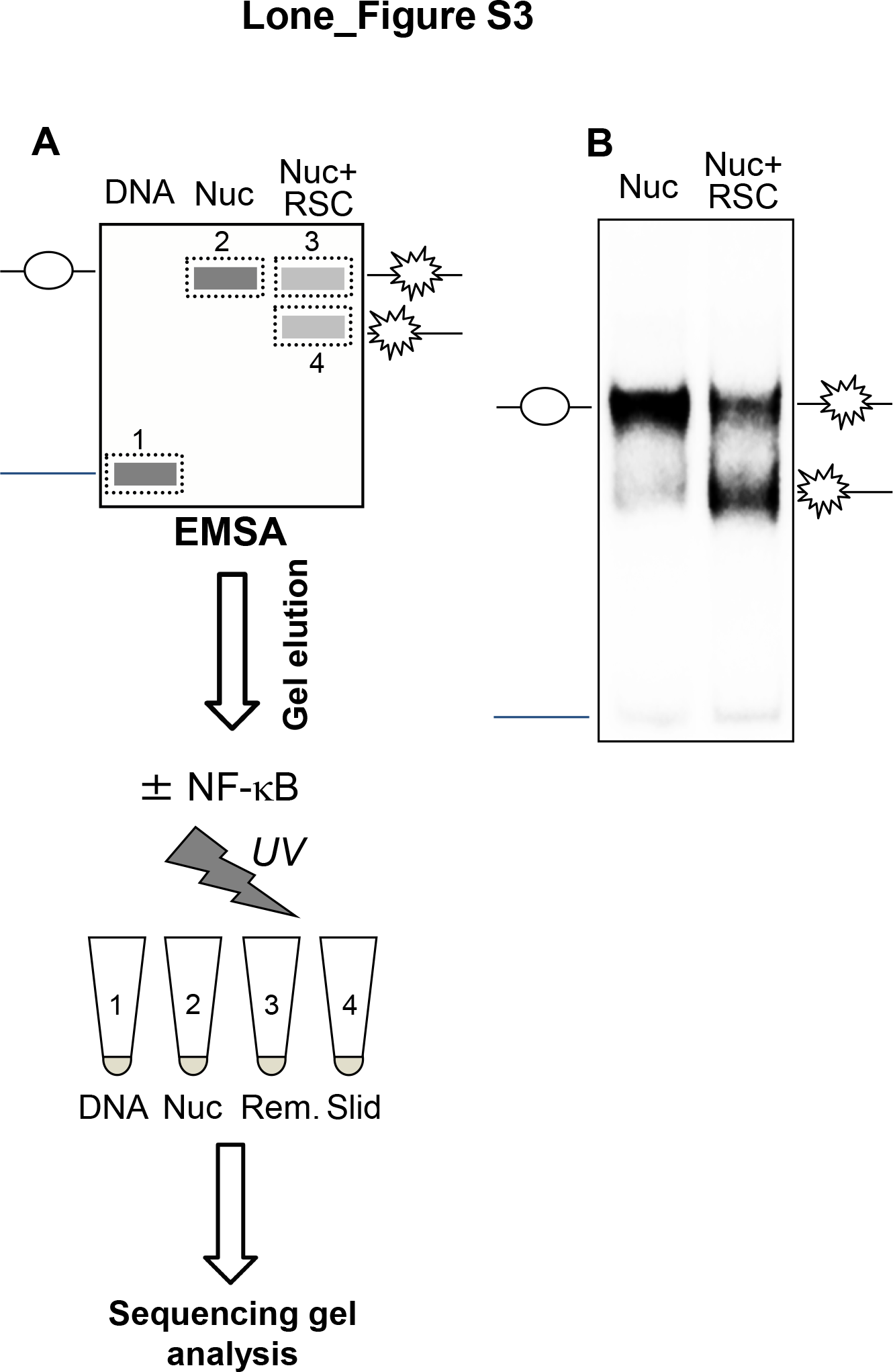

Supplement: Figure S3 — Remosome purification assay. (A) Schematic: Naked DNA, nucleosomes and the remodeling reaction products (remosomes and slid nucleosomes) were run on 5% polyacrylamide gel. The corresponding bands (1, 2, 3, and 4) were cut and the substrates were eluted from the gel as described in material and methods. The purified substrates were then allowed to bind saturating amount of NF-κB (100 nM for DNA and 400 nM for other substrates) and submitted to UV laser footprinting. The formation of complexes with NF-κB is analyzed by 5% polyacrylamide gel. (B) The original preparative 5% PAGE. (TIF) [file pgen.1003830.s003.tif]

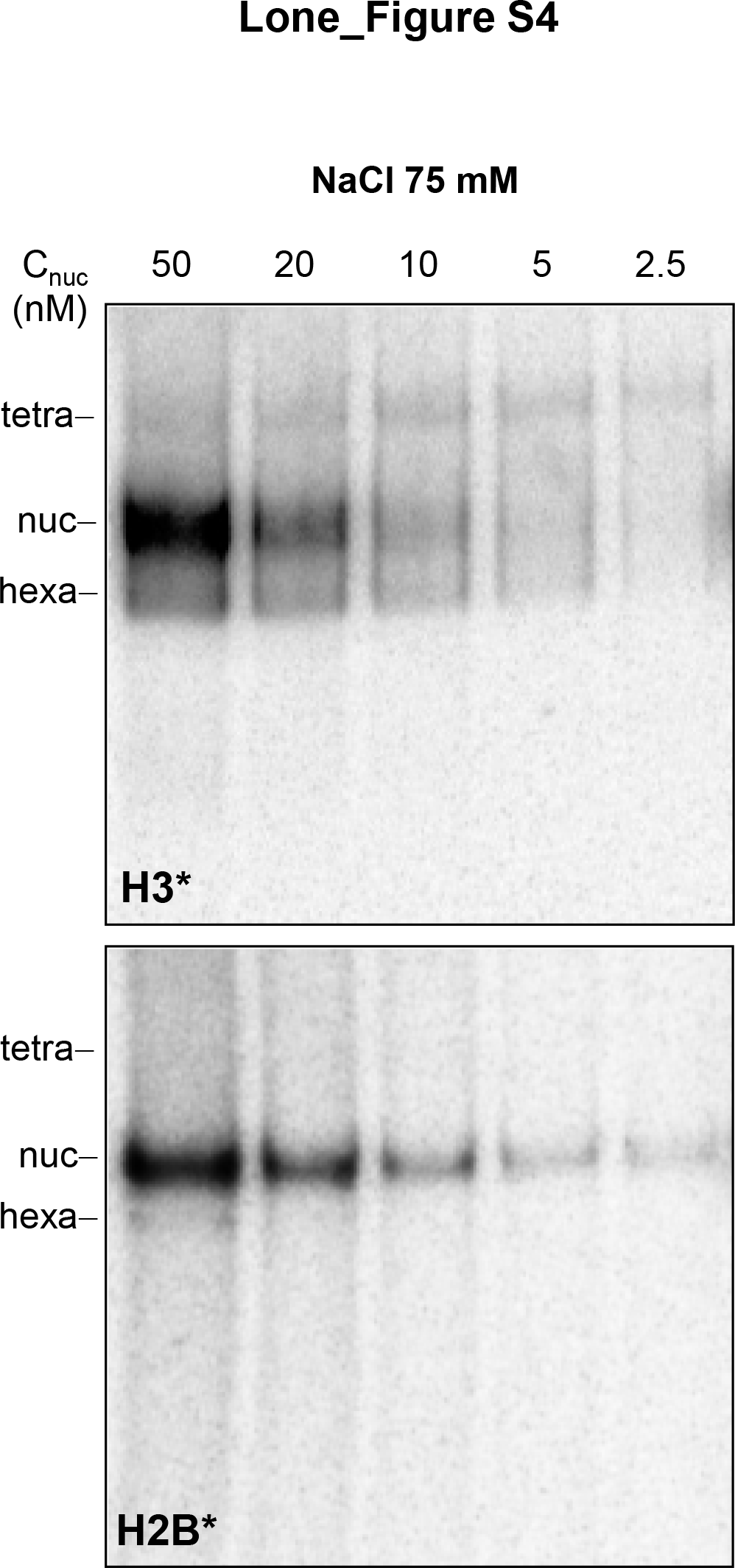

Supplement: Figure S4 — H2A/H2B dimer loss upon nucleosome dilution. Nucleosome dilution experiment was performed by using radioactive labeled histone H3* and H2B* by Aurora kinase B and [γ-32P]ATP as described in [47]. These labeled histones together with other recombinant histones were used to reconstitute H2B* labeled or H3* labeled nucleosomes. Aliquots of nucleosomes were diluted with the appropriate buffer (75 mM NaCl) in a 20 µl final volume to the concentrations indicated (from 50 to 2.5 nM) and left for 45 min at room temperature. Then the samples were analyzed by electrophoretic mobility shift assay carried out in 5% polyacrylamide gel in 0.3× TBE at 4°C. (TIF) [file pgen.1003830.s004.tif]

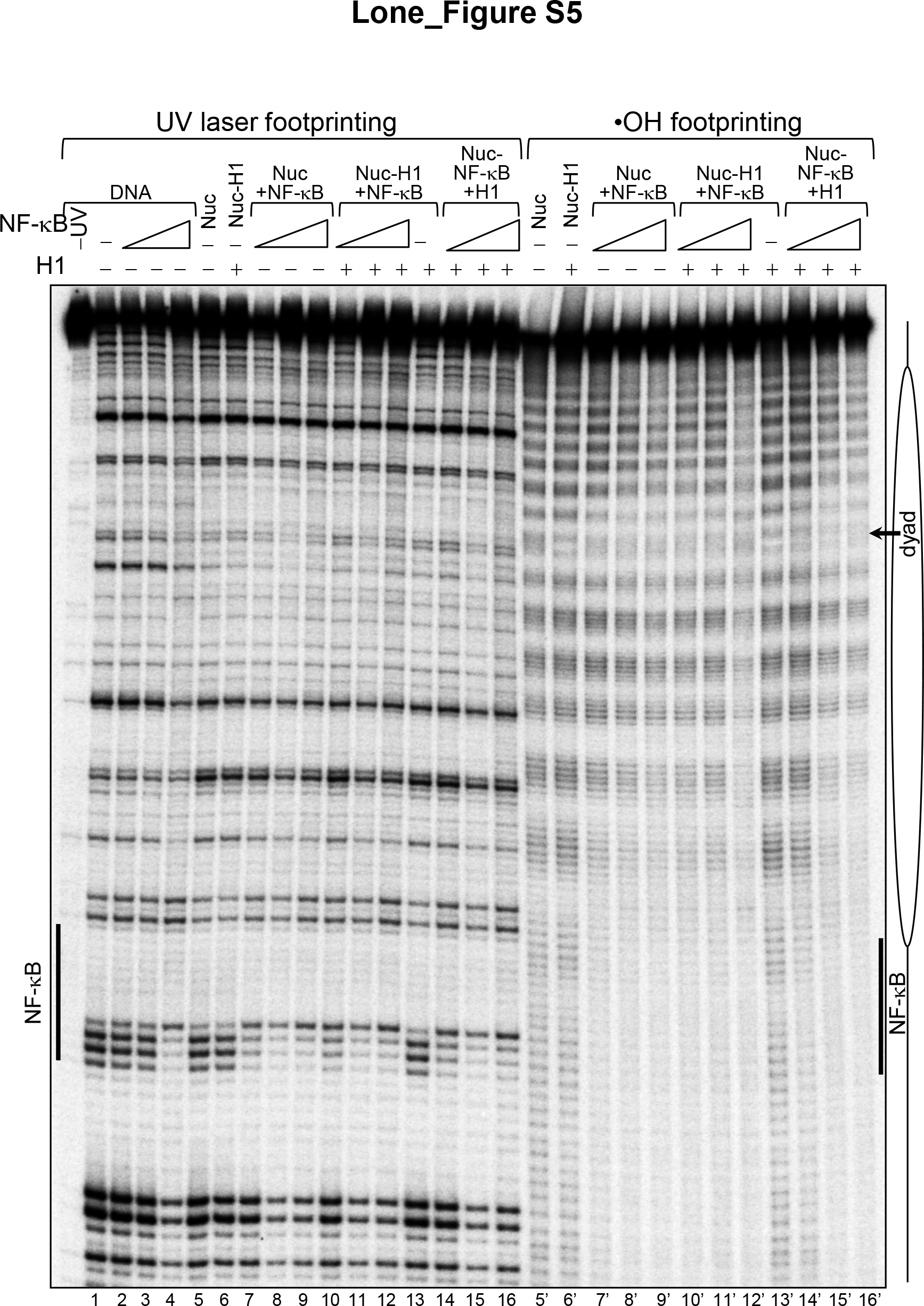

Supplement: Figure S5 — Hydroxyl radical and UV laser footprinting of NF-κB–DNA/nucleosome/chromatosome complexes. 255 bp 601_D8 DNA was 32-P end labeled and used to reconstitute centrally positioned nucleosomes. Chromatosomes were assembled by using the NAP-1/H1 complex to deposit H1 on the nucleosome under “physiological” conditions. Complete gel of the experiment shown in figure 5 for the overall comparison and analysis of UV laser footprinting (lanes 1–16) and •OH footprinting (lanes 5′–16′) (for details see figure 5). (TIF) [file pgen.1003830.s005.tif]

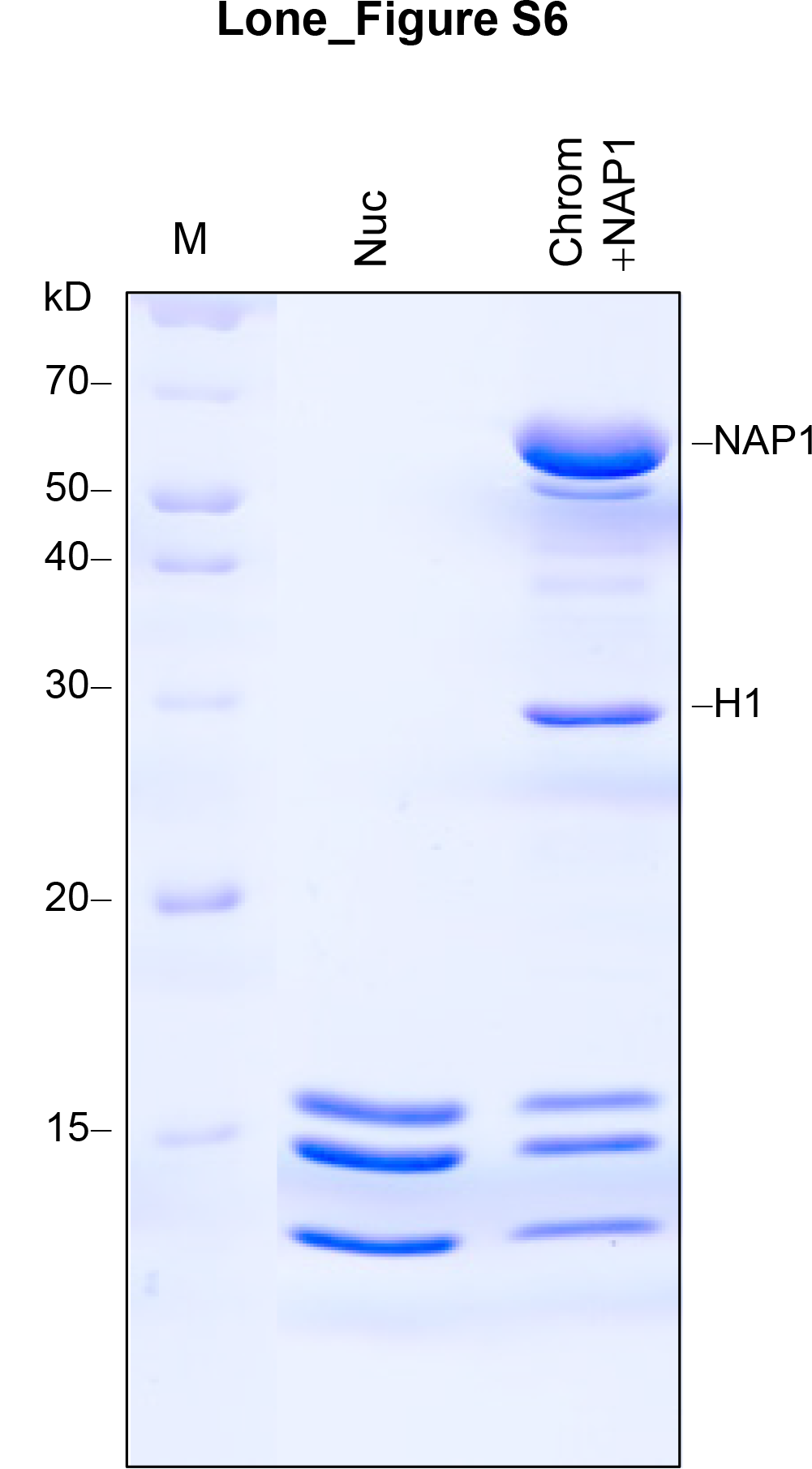

Supplement: Figure S6 — SDS PAGE of nucleosomes and chromatosomes. Reconstituted nucleosomes and chromatosomes were analyzed on 18% SDS gel for verifying the histone composition after the buffer exchange for hydroxyl radical footprinting. (TIF) [file pgen.1003830.s006.tif]
